# A Metabolic Labeling Strategy for Relative Protein Quantification in *Clostridioides difficile*

**DOI:** 10.3389/fmicb.2018.02371

**Published:** 2018-10-16

**Authors:** Anke Trautwein-Schult, Sandra Maaß, Kristina Plate, Andreas Otto, Dörte Becher

**Affiliations:** Department of Microbial Proteomics, Institute of Microbiology, University of Greifswald, Greifswald, Germany

**Keywords:** *Clostridioides difficile*, mass spectrometry, metabolic labeling, proteomics, relative protein quantification

## Abstract

*Clostridioides difficile* (formerly *Clostridium difficile*) is a Gram-positive, anaerobe, spore-forming pathogen, which causes drug-induced diseases in hospitals worldwide. A detailed analysis of the proteome may provide new targets for drug development or therapeutic strategies to combat this pathogen. The application of metabolic labeling (ML) would allow for accurate quantification of significant differences in protein abundance, even in the case of very small changes. Additionally, it would be possible to perform more accurate studies of the membrane or surface proteomes, which usually require elaborated sample preparation. Such studies are therefore prone to higher standard deviations during the quantification. The implementation of ML strategies for *C. difficile* is complicated due to the lack in arginine and lysine auxotrophy as well as the Stickland dominated metabolism of this anaerobic pathogen. Hence, quantitative proteome analyses could only be carried out by label free or chemical labeling methods so far. In this paper, a ML approach for *C. difficile* is described. A cultivation procedure with ^15^N-labeled media for strain 630Δ*erm* was established achieving an incorporation rate higher than 97%. In a proof-of-principle experiment, the performance of the ML approach in *C. difficile* was tested. The proteome data of the cytosolic subproteome of *C. difficile* cells grown in complex medium as well as two minimal media in the late exponential and early stationary growth phase obtained via ML were compared with two label free relative quantification approaches (NSAF and LFQ). The numbers of identified proteins were comparable within the three approaches, whereas the number of quantified proteins were between 1,110 (ML) and 1,861 (LFQ) proteins. A hierarchical clustering showed clearly separated clusters for the different conditions and a small tree height with ML approach. Furthermore, it was shown that the quantification based on ML revealed significant altered proteins with small fold changes compared to the label free approaches. The quantification based on ML was accurate, reproducible, and even more sensitive compared to label free quantification strategies.

## Introduction

*Clostridioides difficile* (Lawson et al., 2016), formerly known as *Clostridium difficile* (Hall and O’Toole, 1935), is an ubiquitous, obligate anaerobic, spore forming, Gram-positive bacterium with a close relation to the *Peptostreptococcaceae* family (Collins et al., 1994; Yutin and Galperin, 2013). *C. difficile* is the leading cause of healthcare-associated infective diarrhea (Freeman et al., 2010) with approximately 500,000 patients in the United States (Lessa et al., 2015) and 124,000 patients in the European Union in 2011 (Smits et al., 2016). The main risk factor for the CDI is the antibiotic exposure during hospitalization or the association of patients with the health care facility (Freeman et al., 2010; Lessa et al., 2015). In 2006, the total costs of CDI were evaluated at 3 billion EURO per year in the European Union with an increasing tendency (Kuijper et al., 2006). The mortality rate caused by CDI has been reported to be approximately 10% within a month of diagnosis leading to approximately 29,000 deaths in the United States in 2011 (Planche et al., 2013; Lessa et al., 2015).

Since the first description of *C. difficile* as cause of antimicrobial-associated diarrhea in humans and animals, the scientific interest in this pathogen increased rapidly. Epidemiological studies described numerous strains around the world (Freeman et al., 2010) and an alarming number of antimicrobial resistances for several classes of antimicrobial agents (Spigaglia et al., 2011). Due to the increased necessity of profound knowledge about the pathogenicity, pathophysiology, virulence factors, or resistance mechanisms of *C. difficile*, several proteomic studies of this pathogen were performed in the last two decades. In addition to a variety of other studies, the publications on cell wall associated proteins and proteins of the spore layers are particularly interesting. Cell wall associated proteins of the pathogen interact with the host and the microbiota and could be potential targets for the immune system or antimicrobial agents while spores play an important role for the infection as well as during spreading processes and antibiotic resistance. Wright et al. (2005) identified cell wall associated proteins (two surface layer proteins, components of the flagella, and a number of paralogs of the high-molecular weight surface layer protein) in a 2D SDS-PAGE approach after different extraction methods. In further studies, proteins of different spore layers involved in the spore coat morphogenesis, in the attachment to surfaces, or proteins with a possible role in spore resistance or germination were identified (Abhyankar et al., 2013; Swarge et al., 2018).

Chen et al. (2017) reviewed different proteomic approaches of the recent years which have been used for discovery of host–pathogen interactions, which were observed and described on different levels: (i) molecular level (identification of microbial virulence proteins and protein modifications), (ii) single-cell level (exploration of microbial resistance mechanisms), (iii) organism level (non-invasive body fluid analysis for diagnostic and prognostic biomarker identifications), and (iv) population level (studying the gut microbiome and metaproteome). Such in-depth analysis of *C. difficile* would offer new starting points for the identification of targets for therapeutic strategies. Especially, detailed analyses of the membrane and surface proteome of *C. difficile* are essential for a better understanding of, e.g., virulence associated processes, ways of infection as well as resistance mechanisms of one- or multi-drug-resistant strains. The detailed knowledge about the membrane proteome of *C. difficile* could lead to the identification of protein candidates as vaccine targets at the cell surface like previously described for *Francisella tularensis*. The proteome analysis of the inner and outer membrane of *F. tularensis* showed two additional proteins in an attenuated strain compared to a virulent strain. The membrane of the attenuated strain was used as vaccination and led to an significant protection against the virulent strain (Post et al., 2017).

For quantitative analysis of adaptation processes on proteome level, various techniques are described, which are based either on the introduction of stable isotopes into proteins or peptides or on determination of protein amounts in a label free manner. Each of these techniques owns specific advantages and inevitable disadvantages (Bantscheff et al., 2007, 2012; Otto et al., 2012; Chahrour et al., 2015). In general, the main advantages of the label based approaches are a high accuracy and reproducibility whereas the label free approaches show a higher quantitative proteome coverage and can be used for the proteomic analysis of each organism (Bantscheff et al., 2007). Since the first description of a stable isotope labeling introduced by ML for bacterial proteomes (Oda et al., 1999), the labeling strategy was adapted also for cell cultures with the development of SILAC by Ong et al. (2002). The application of ML offers the introduction of a stable isotope label into each protein at the earliest time point, during protein synthesis. The combination of unlabeled (‘light’) and labeled (‘heavy’) proteins during the first steps of a proteome analysis workflow (Oda et al., 1999; Ong et al., 2002) allows for correction of all sources of quantification errors possibly introduced during sample preparation (Bantscheff et al., 2007). Relative protein quantification is performed by the comparison of the abundance of precursor ions in the survey spectrum derived from co-eluting ‘heavy’ and ‘light’ peptides (Oda et al., 1999). Due to the early combination of differentially labeled samples, the ML approach is supposed to be the most accurate label based quantitative MS method which makes it particularly suitable for detecting small changes in protein amounts (Bantscheff et al., 2007). In addition to relative quantitative studies, ML approaches can also be used for determination of protein turnover for individual proteins as shown for the first time for 50 selected proteins of *Saccharomyces cerevisiae* with an average degradation rate of about 2.2% per hour (Pratt et al., 2002). This knowledge about individual protein synthesis and degradation rates becomes essential for integrated Omics techniques (Beynon and Pratt, 2005). Another possible application of the ML approach offers the opportunity to quantify secreted proteins from *C. difficile* which are redundant in the secretome of the surrounding bacterial consortium. A similar approach used azidonorleucine based ML strategy of *Yersinia enterocolitica* before infection of HeLa cells to study low abundant microbial proteins in the host cell cytoplasm (Mahdavi et al., 2014).

Until now, protein quantification in *C. difficile* was based on chemical labeling of peptides via iTRAQ (Jain et al., 2011; Chong et al., 2014) or TMT labeling technology (Chen et al., 2013) as well as on LFQ (Chilton et al., 2014; Dresler et al., 2017). Additionally, the first global analysis of the lipoproteome of different *C. difficile* strains was performed using metabolic tagging with an alkyne-tagged fatty acid analog for enrichment of lipoproteins and quantitative label free proteomic profiling (Charlton et al., 2015). The highest number of quantified proteins (1.578) was achieved applying a LFQ approach, which was employed to disclose physiological differences of *C. difficile* in the same growth phase (exponential growth phase) but different growth media (BHI medium and CDMM) (Otto et al., 2016). Despite the high number of quantified proteins included in this study, only 24% of the approximately 1000 proteins, predicted to be localized in the *C. difficile* membrane, were identified. Hence, for the analysis of adaptation processes in the membrane or the cell surface on proteome level, a subcellular fractionation and enrichment procedures would lead to an increased number of membrane proteins.

For a ML of each tryptic peptide (except of the C-terminal peptide), the organisms should be arginine and lysine auxotroph (Ong et al., 2002). Unfortunately, *C. difficile* has only a proline, cysteine, leucine, isoleucine, tryptophan, and valine auxotrophy (Karasawa et al., 1995). Additionally, the metabolism of *C. difficile* is dominated by the Stickland reaction (Bouillaut et al., 2013; Neumann-Schaal et al., 2015), which complicates the whole ML process. As shown for *Bacillus subtilis* (Otto et al., 2010), an *in vivo* ^15^N ML approach could present a suitable alternative to a SILAC ML approach with several selected amino acids for relative protein quantification. Therefore, a ML based on ^15^N incorporation during protein synthesis was established for *C. difficile* in this study. In the result, the first proteome study of the obligate anaerobe *C. difficile* using a ML approach is reported. Here, the quantitative changes in protein abundances were compared in three different media in late exponential and early stationary growth phase by using ML for label based quantification as well as NSAF based on spectral counting and LFQ based on peak intensities as LFQ methods. The software packages used in this work were chosen according to the quantification strategy. For ML with ^15^N only a few software packages are available. Census, the used software, is based on a SEQUEST search. Therefore, the first choice for the label free quantification was based on SEQUEST search as well and further processes to NSAF quantification which is often used in the proteomics community for label free quantification. To take the rapid growing community of MaxQuant users into account, MaxQuant LFQ was used as second quantification method. Finally, the performance of the three quantification approaches was compared.

## Materials and Methods

### Bacterial Strain, Spore Purification and Culture Conditions

*Clostridioides difficile* 630Δ*erm* (Hussain et al., 2005; Lawson et al., 2016) was obtained from the Deutsche Sammlung von Mikroorganismen und Zellkulturen (DSMZ, Germany). Three different media were used: BHI medium (37 g/l BHI, Oxoid) supplemented with resazurin (1 mg/l) as oxygen indicator, CDMM (Neumann-Schaal et al., 2015) and CDCM, as described in detail in **Supplementary Table S1**. ^15^N-CDCM was prepared with ^15^N-labeled Celtone and ^15^N-labeled ammonium sulfate (98%, Cambridge Isotope Laboratories, United States). After autoclaving or preparation of sterile media, the media were gassed with oxygen-free gas. The reduced media as well as all consumables were stored overnight in the anaerobic chamber before use (Uchino and Ken-Ichiro, 2013).

For storage of the strain and reproducible start of cell culture, *C. difficile* spores were used. In order to produce and purify spores, a single *C. difficile* colony from fresh pre-reduced BHIS plates [BHI medium supplemented with 0.5% (w/v) yeast extract, 1.5% (w/v) agar and after autoclaving 0.1% (w/v) L-cysteine] was grown in 20 ml BHI in an anaerobic chamber (5% H_2_, 95% N_2_) at 37°C for at least 2 days. Spores were purified as previously described (Edwards et al., 2013), stored at 4°C, and incubated at 55°C for 15 min before inoculation of a culture to enable efficient germination.

Spores germinated in BHI medium supplemented with 0.1% (w/v) sodium taurocholate at 37°C for at least 16 h. Cells were passaged with a 1:100 dilution in CDMM after at least 16 h. The cultivation of the main cultures in triplicates for the three different media was inoculated to an OD at 600 nm of ∼0.05. Culture growth was monitored by measurement of the OD vs. non-inoculated medium. The complete cultivation took place within the anaerobic chamber to exclude oxygen entry.

### Metabolic Labeling of *C. difficile*

**FIGURE 1:**
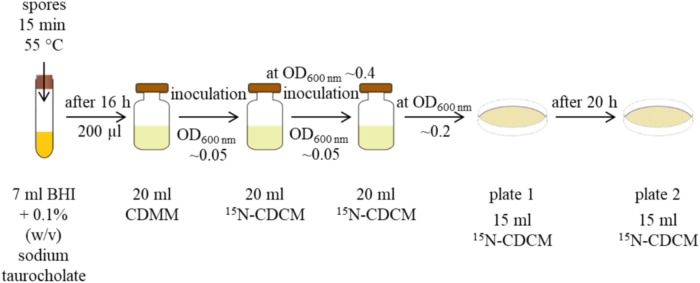
Workflow for preparation of metabolic labeled cells. Spores were heated 15 min at 55°C for efficient germination in BHI medium supplemented with 0.1% (w/v) sodium taurocholate. After at least 16 h cells were passaged with a 1:100 dilution in CDMM and cultured for at least 16 h. These cells were used for inoculation of liquid ^15^N-CDCM to an OD_600 nm_ ∼0.05. Cells from early exponential growth phase (OD_600 nm_ of 0.4) were used to inoculate fresh liquid ^15^N-CDCM to an OD_600 nm_ ∼0.05. Cultures with an OD_600 nm_ ∼0.2 were plated on ^15^N-CDCM and cultured. Cells grown for 20 h on plates were washed with 1× PBS, scraped off with a cell scraper, and used for inoculation of new ^15^N-CDCM plates. After 20 h cells were washed with 1× PBS and harvested with a cell scraper.

Germinated spores were passaged from BHI to CDMM. Cells from the CDMM culture were used for inoculation of liquid ^15^N-CDCM to an OD_600 nm_ of ∼0.05. Cells from early exponential growth phase (OD_600 nm_ of 0.4) were used to inoculate fresh liquid ^15^N-CDCM to an OD_600 nm_ of ∼0.05. After two additional cell divisions in liquid media, cells were plated on fresh pre-reduced ^15^N-CDCM plates, cultured for 20 h at 37°C. Cells were washed with phosphate buffered saline (1× PBS) (8 g/l NaCl, 0.2 g/l KCl, 1.44 g/l Na_2_HPO_4_, 0.27 g/l KH_2_PO_4_, pH 7.4 HCl) and scraped off with a cell scraper. This complete cultivation step on plates was repeated. The workflow for ML procedure is summarized in **Figure 1**.

### Cell Harvest

In late exponential growth phase (OD_600 nm_ of 0.8 to 1.0) and early stationary growth phase (2 h after constant or maximal OD_600 nm_) 10 ml of each culture were harvested by centrifugation (5 min, 5.000 × *g*, 4°C) and washed twice with 1× PBS. These samples were named ‘light sample’. Similarly, the labeled cells were washed with 1× PBS and scraped off with a cell scraper, harvested by centrifugation (10 min, 5.000 × *g*, 4°C) and washed twice with 1× PBS. These samples were named ‘heavy standard.’

### Protein Preparation

Cell pellets were suspended in 400 μl lysis buffer (50 mM Tris, 10 mM EDTA, 70 mM DTT, pH 7.4) and lysed by ultrasonication at 4°C (Bandelin Sonoplus HD 3200 with MS73, 6 cycles of 1 min 70% amplitude). Cell debris was removed by centrifugation (45 min, 20.000 × *g*, 4°C) and the supernatant was kept as cytosolic protein fraction. Protein concentrations of extracts were determined using the Bradford assay with bovine serum albumin as standard (Bradford, 1976).

Twenty μg of light sample protein (^14^N) was used for LFQ whereas 10 μg of light sample protein spiked with 10 μg of heavy standard protein was used for quantification based on ML. Protein samples were supplemented with loading buffer and heated for 5 min at 95°C before separation via SDS-PAGE (Criterion TG 4-20% Precast Midi Gel, BIO-RAD Laboratories, Inc., United States). As previously described (Bonn et al., 2014), after staining, each gel lane was cut into pieces, destained, desiccated and rehydrated in trypsin. In gel-digest was incubated at 37°C overnight. Peptides were eluted with water by sonication for 15 min and dried in a vacuum centrifuge.

### Liquid Chromatography and Mass Spectrometric Analysis

Just before MS analysis, the indexed retention time standard kit (Biognosys, Switzerland) was prepared according to manufacturer’s instructions and was added to each sample in a 1:100 ratio. Peptides were dissolved in 0.1% (v/v) acetic acid and loaded on an EASY-nLC II (Thermo Fisher Scientific, United States) system equipped with an in-house built 20 cm column (inner diameter 100 μm, outer diameter 360 μm) filled with ReproSil-Pur 120 C_18_-AQ reversed-phase material (3 μm particles, Dr. Maisch GmbH, Germany). As previously described (Otto et al., 2016), elution of peptides was executed with a non-linear 80 min gradient from 1 to 99% solvent B (0.1% (v/v) acetic acid in acetonitrile) with a flow rate of 300 nl/min and injected online into a LTQ Orbitrap Velos Pro (Thermo Fisher Scientific, United States). The survey scan at a resolution of *R* = 30.000 and 1 × 10^6^ automatic gain control target in the Orbitrap with activated lock mass correction was followed by selection of the 20 most abundant precursor ions for fragmentation. Singly charged ions as well as ions without detected charge states were excluded from MS/MS analysis. All MS data have been deposited to the ProteomeXchange Consortium via the PRIDE partner repository (Vizcaíno et al., 2016) with the dataset identifier PXD010279.

### Analysis of Metabolic Labeling Data

The workflow for analysis of ML data was adopted from Otto et al. (2010). Briefly, ^∗^.raw files were searched with Sorcerer SEQUEST v. 27, rev. 11 (Thermo Fisher Scientific, United States) against a *C. difficile* 630Δ*erm* database (Dannheim et al., 2017) containing 7646 protein entries (common laboratory contaminations and all reversed sequences added). The search was performed in two iterations using full digest with trypsin (after KR/); 10 ppm peptide mass tolerance; variable modification methionine oxidation (15.99 amu); and for the second iteration, mass shift of all amino acids completely labeled with ^15^N-nitrogen. Resulting ^∗^.dta and ^∗^.out files from both searches were assembled and filtered using DTASelect 2.0.25. MS1 mass traces were extracted from the ^∗^.raw files with RawXtract 1.9.9.2. The processed results were analyzed using Census 1.72 (Park et al., 2008) to obtain quantitative data of ^14^N-peaks (light sample) and ^15^N-peaks (heavy standard). Proteins that failed to be relatively quantified were checked and, if reasonable, edited manually in the graphical user interface of Census. Relative quantification data for proteins with at least two quantified unique peptides were exported (*R*^2^ > 0.7) and used for subsequent analysis. Quantitative ratios were log_2_-transformed, normalized by central median tendency, and used for statistical analysis.

### Evaluation of Incorporation Rates

For the evaluation of the incorporation rates, 10 μg light sample was spiked with 10 μg heavy standard and separated via SDS-PAGE. One small piece of the SDS-Gel was destained and in gel-digested. The resulting peptides were used for LC-MS/MS process as described before. The search was performed in two iterations considering that the light and heavy labeled proteins are analyzed. From the resulting list of proteins, 10 peptides were chosen which could be identified with both database searches. The freeware tool ‘IDCalc – Isotope Distribution Calculator’ provided by the MacCoss laboratory at University of Washington^1^ allows the assessment of the incorporation rate (Otto, 2018).

### Analysis of Label Free Data – Normalized Spectral Abundance Factors

For calculation of NSAF (Zybailov et al., 2006), the ^∗^.raw files were searched with Sorcerer SEQUEST v. 27, rev. 11 as described above. Scaffold (v. 4.7.5, Proteome Software Inc., United States) was used to validate MS/MS based peptide and protein identifications. Proteins were only considered as identified if at least two unique peptides matched solid quality criteria (deltaCn > 0.1 and XCorr > 2.2; 3.3; 3.75 for doubly, triply, or higher charged peptides, respectively). Protein quantification was based on NSAFs, which are calculated as the number of spectral counts (SpC) identifying a protein, divided by protein length (L), divided by the sum of SpC/L for all proteins in the experiment using Scaffold’s exclusive spectrum counts for each protein. NSAFs were log_2_-transformed and used for statistical analysis.

### Analysis of Label Free Data – LFQ Intensity

For LFQ with MaxQuant software (v. 1.6.0.16) (Cox et al., 2014), the ^∗^.raw files were searched against a *C. difficile* 630Δ*erm* database with MaxQuant’s generic contamination list included. Database search was performed using the Andromeda algorithm with following parameters: digestion mode, trypsin/P with up to 2 missed cleavages; variable modification, methionine oxidation, and maximal number of 5 modifications per peptide; activated LFQ option with minimal ratio count of 2 and ‘match-between-runs’ feature. The false discovery rates of peptide spectrum match and protein were set to 0.01. Only unique peptides were used for protein quantification. The data from MaxQuant output files were filtered for contaminants, only identified by site and reverse hits with the Perseus software (v. 1.6.1.1), log_2_-transformed and used for statistical analysis.

### Further Data Analysis and Statistical Analysis

A protein was considered to be identified if two or more unique peptides were identified in a biological replicate. Proteins were considered to be quantified if a quantitative value based on at least two unique peptides was available in at least two biological replicates. For calculation of logarithmic fold change with base 2 (log_2_FC), the log_2_-transformed normalized quantitative ratios were averaged over the three biological replicates. The calculated log_2_FC was referred to either the data obtained for exponential growing cells in BHI (in case of exponential growth phase samples in CDMM and CDCM) or to data derived from stationary growth phase cells in BHI (in case of stationary growth phase samples in CDMM and CDCM).

The CV per protein over three biological replicates was calculated using square root transformed ML ratios, NSAF values, and LFQ intensities, whereas the ratios were median normalized additionally. The two-factor ANOVA analysis was carried out using TMEV (v. 4.9.0) on protein level. Statistical significance required a *p*-value < 0.01. Treemaps of the *C. difficile* 630Δ*erm* proteome were built using the Paver software (DECODON GmbH, Germany) (Bernhardt et al., 2009) on the basis of TIGR roles assigned according to Otto et al. (2016). Violin plots were prepared using the software environment R (v. 3.5.0) (R Core Team, 2017).

## Results

### ^15^N Metabolic Labeling in *C. difficile*

The major aim of this study was the establishment and evaluation of a ML procedure for relative quantification of the *C. difficile* proteome. A high incorporation rate is necessary for a successful identification and quantification of proteins in a ML approach. For a protein labeling of more than 90% in media supplemented with stable isotopes like SILAC cells needs to achieve 6–8 passages (Bantscheff et al., 2007). Unfortunately, *C. difficile* is able to grow with amino acids as sole source for carbon or energy via the Stickland reaction (Bouillaut et al., 2013; Neumann-Schaal et al., 2015). Accordingly, a ML with SILAC is no option for *C. difficile* because this pathogen has only proline, cysteine, leucine, isoleucine, tryptophan, and valine auxotrophy (Karasawa et al., 1995). Therefore, the ^15^N ML was used.

Various cultivation procedures were tested to achieve a protein labeling with ^15^N of at least 90%. In liquid ^15^N-labeled CDCM, *C. difficile* cells grew up to a maximal OD_600 nm_ of 1.5 followed by lysis of the cells. As expected, the incorporation rate of extracted proteins after 5 passages in media supplemented with stable heavy isotopes proved to be insufficient for quantitative analysis. In order to increase the incorporation rate of the stable isotopes by labeling over more generations, it was necessary to use pre-cultures with media containing stable isotopes as well. Cells growing in ^15^N-substituted CDCM were transferred to fresh liquid ^15^N-CDCM. Unfortunately, the ability to grow for a second time in liquid ^15^N-CDCM was dramatically reduced (data not shown). To reach a sufficient incorporation rate, various cultivations strategies were carried out in liquid and solid medium as summarized in **Supplementary Figure S1**. Only with the combination of culturing in liquid ^15^N-substituted CDCM and solid ^15^N-CDCM an average incorporation rate of 97.6% of ^15^N could be achieved (**Figure 1**).

### Comparison of Protein Identification and Quantification and Reproducibility of the Quantification Approaches

After the successful establishment of the ML approach, the performance of this labeling approach in *C. difficile* was compared with two label free relative quantification strategies (NSAF and LFQ). For this comparison, protein extracts of *C. difficile* 630Δ*erm* cells grown in complex media (BHI) as well as minimal media (CDMM and CDCM) in the late exponential and early stationary growth phase were analyzed by LC-MS/MS for three biological replicates. The workflow of the proteomic experiment is illustrated in **Supplementary Figure S2**.

For quantitative protein analysis based on ML approach the resulting LC-MS/MS data were searched against a *C. difficile* 630Δ*erm* specific database using the Sorcerer SEQUEST platform. In summary, 53% of the theoretical proteome was identified in at least one replicate (2,020 proteins). For approximately half of the identified proteins the signal quality was sufficient for quantification resulting in 1,110 quantified proteins in at least two replicates (**Table 1**). The median SD of the median normalized ratios for all quantified proteins was 0.076. The detailed data of identified and quantified proteins are listed in **Supplementary Table S2**.

**Table 1:** Listed number of identified, quantified, and significantly changed proteins with ML, NSAF, and LFQ approaches.

	ML	NSAF	LFQ
Numbers of identified proteins	2,020	1,788	2,019
Number of quantified proteins	1,110	1,545	1,861
Number of significantly changed proteins	322	365	610


*Proteins were identified with two unique peptides in one biological sample and quantified with at least two unique peptides in at least two of the three biological replicates. Significantly changed proteins were determined with two-factor ANOVA due to growth phase, media or both factors and counted with a p-value < 0.01 in at least one of the three analysis.*

For the two LFQ approaches the same LC-MS/MS raw data and the same *C. difficile* 630Δ*erm* specific database were used as shown in the summarized workflow in **Supplementary Figure S2**. For quantification based on NSAFs MS data were searched using the Sorcerer SEQUEST platform which generated Scaffold output files. In Scaffold NSAF values for 1,788 identified proteins were calculated of which 1,545 proteins could be considered for quantification as NSAF-values of at least two biological replicates were determined (**Table 1**). The median SD of the median normalized number of spectra was 0.125 indicating a higher deviation compared to the ML approach.

As an alternative LFQ strategy, the LFQ strategy provided in the MaxQuant software was used. Here the database search as well as the quantification is performed in MaxQuant. In this approach total of 2,019 proteins were identified and 1,861 of these proteins were quantified in at least two biological replicates (**Table 1**). The median SD of the median normalized peak areas was 0.124 and therefore in the same range as in the NSAF approach.

**FIGURE 2:**
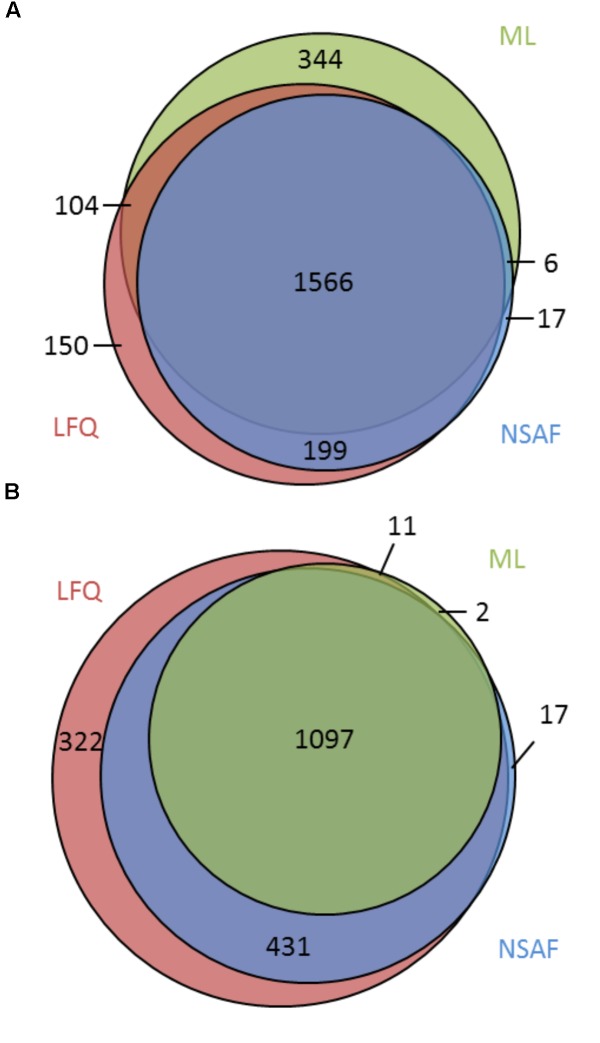
Comparison of qualitative and quantitative results obtained from different approaches. **(A)** Overlap of identified proteins using the ML approach or label free approaches, NSAF, and LFQ. **(B)** Overlap of quantified proteins using the ML, NSAF, or LFQ approaches.

Comparing the three approaches, it becomes apparent that the majority of 1,566 (65.6%) proteins were identified with all approaches, whereas 344 (14.4%), 17 (0.7%), and 150 (6.3%) proteins were identified exclusively in the ML, NSAF, or LFQ approach, respectively (**Figure 2A**). With respect to quantification the majority of 1,097 (58.4%) proteins were quantified within all three approaches, whereas two proteins (0.1%), 17 proteins (0.9%), and 322 proteins (17.1%) were exclusively quantified with the ML, NSAF or LFQ approach, respectively (**Figure 2B**).

**FIGURE 3:**
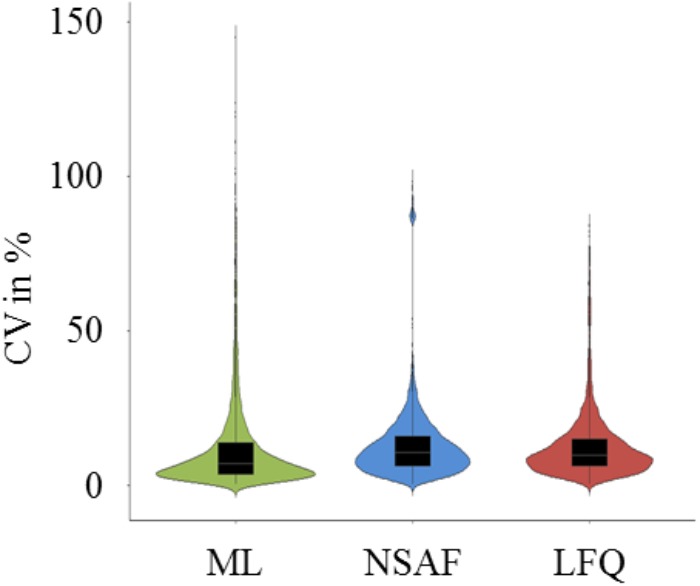
Violin plots of CV distribution. Calculated CV of the normalized, square root transformed sample/standard ratios (ML approach), square root transformed number of spectra (NSAF approach), and square root transformed peak areas (LFQ approach) from 539 quantified proteins of the two growth phases and the three media for each quantification approach were incorporated to one plot for ML (green violin), NSAF (blue violin), and LFQ (red) based quantification.

In order to compare the quantification results based on ML, NSAF as well as LFQ, the dataset was reduced to proteins which were quantified in all six growth conditions (complex medium and two minimal media; in late exponential and early stationary growth phase) and in all three quantification approaches. The resulting 539 quantified proteins were used for further comparison analysis. The CV for these 539 proteins were calculated for each condition of the three quantification approaches (**Supplementary Table S3**) and the distribution of the CV is depicted in **Figure 3**. The CV distribution of ML based quantification was scattered over a wider range but the majority of the CVs were lower than 15% with a median of 6.78%, whereas the CV distribution for the LFQ approaches were slightly shifted upward with a median of 10.30% and 9.56% for NSAF and LFQ approach, respectively.

**FIGURE 4:**
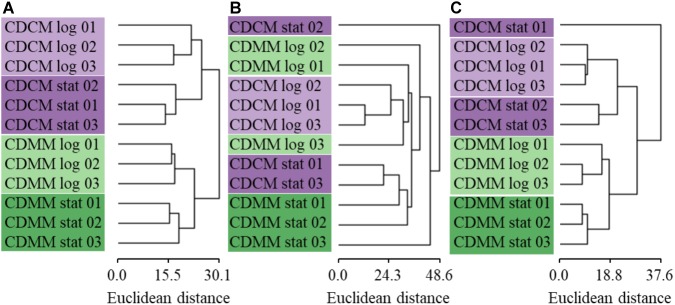
Hierarchical clustering analysis of 539 proteins of **(A)** ML, **(B)** NSAF, and **(C)** LFQ quantification approaches was performed using the log_2_FC values for each biological replicate growing in CDCM (purple) or CDMM (green) either in late exponential (log, light color) or early stationary growth phase (stat, dark color) referred to the averaged log_2_FC data obtained either for late exponential growth phase in BHI or data derived from early stationary growth phase in BHI.

To compare the quantitative values of the three quantitative approaches (sample/standard ratios, normalized number of spectra, normalized peak areas) the log_2_FC was calculated. Therefore the log_2_-transformed normalized quantitative values were averaged over the three biological replicates. The calculated values of BHI (late exponential as well as early stationary growth phase) were used as reference for the calculation of the log_2_FC in case of late exponential growth phase samples in CDMM and CDCM or in case of early stationary growth phase samples in CDMM and CDCM. A HCL analysis was performed for all 539 proteins based on the log_2_FC of all three quantification approaches (**Supplementary Table S4**). For ML approach the HCL showed clearly separated clusters of the biological replicates between different conditions as well as a small tree height including low total variation between the data. In contrast, the clustering of the LFQ approaches had a less definite resolution and showed a higher tree height, whereas NSAF performed worse than LFQ based approach (**Figure 4**).

### Correlation of Protein Quantification

**FIGURE 5:**
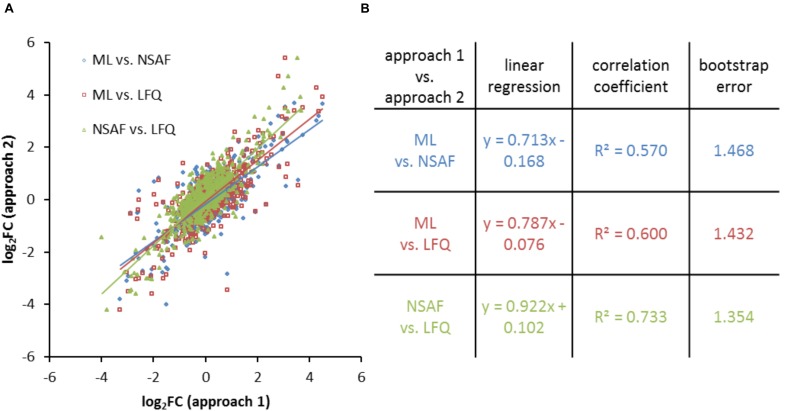
Comparison of quantitative results obtained from different quantification approaches. **(A)** Scatter plot of log_2_FC values obtained from different quantification methods for proteins of CDCM in late exponential growth phase. **(B)** Respective correlation analysis based on linear regression, the correlation coefficient for each linear regression, and the bootstrap error.

As described above, the dataset of the quantified proteins was filtered for proteins which were quantified in all conditions for all approaches (**Supplementary Table S5**). The calculated log_2_FC for these selected proteins were used for the respective correlation analysis which is shown exemplarily by a scatter plot obtained from different quantification methods for cells growing in the late exponential growth phase in CDCM (**Figure 5A**). For all three quantification techniques linear correlations were observed with average *R*^2^ values of 0.634. Linear regressions and correlation coefficients for the proteins in late exponential growth phase in CDCM are listed in **Figure 5B**.

The slopes of the linear regression for ML versus label free approaches (NSAF: 0.713, LFQ: 0.787) were smaller compared to NSAF versus LFQ based quantification (0.922). The correlation coefficient indicated a better comparability of the log_2_FC within the label free approaches compared to the comparability between ML and label free approaches. The averaged deviation between two quantification methods (bootstrap error) is smallest between the two LFQ approaches. The results for the comparison of the three approaches for all growth conditions and media are summarized in **Supplementary Figure S3**.

### Sensitivity of Relative Quantification

For evaluation of the sensitivity of the relative quantification approaches, significantly changed proteins with a two-factor ANOVA (*p*-value < 0.01) were used. The adjusted *p*-values for all proteins significantly changed due to growth phase, media, or both factors are summarized in **Supplementary Table S6**.

As already suggested by the different number of quantified proteins, the number of proteins with significantly changed abundance also differed between the three quantification approaches (**Table 1**). Although fewer proteins were quantified within the ML approach, the percentage of the proteins with significantly changed abundance due to growth phase, media, or both factors was comparable to the LFQ quantification approach and even higher than with the NSAF quantification approach (**Table 2**). As described before, the dataset was reduced to 539 proteins, which were quantified in all conditions for all approaches for increasing the comparability. More than 50% of these 539 proteins showed a significant change in protein abundance depending on the used media in the ML approach whereas 39 and 43% of these proteins showed significantly changed abundance for NSAF and LFQ quantification, respectively. As shown in **Table 2**, the total number of significantly changed proteins decreased by the reduction to 539 proteins because some of the significantly changed proteins could not be quantified in all approaches. But the percentage of significantly changed proteins increased for all approaches due to the reduced number of proteins.

**Table 2:** Percentage as well as number of proteins significantly changed in abundance between the tested growth phases (late exponential versus early stationary growth phase), the tested media (BHI, CDCM vs. CDMM) as well as the influence of the used media on adaptation to the growth phase (interaction).

		ML	NSAF	LFQ
Complete dataset	Growth phase	12% (#136)	6% (#100)	11% (#198)
	Media	25% (#282)	19% (#295)	27% (#502)
	Interaction	7% (#78)	5% (#78)	5% (#99)
Reduced dataset^∗^	Growth phase	25% (#133)	14% (#79)	18% (#96)
	Media	51% (#275)	39% (#212)	43% (#233)
	Interaction	14% (#76)	11% (#58)	9% (#48)


**^#^Total number of proteins with significantly changed abundance. ^∗^Denote the filtered dataset with a total of 539 proteins quantified in all conditions for all approaches.**

**FIGURE 6:**
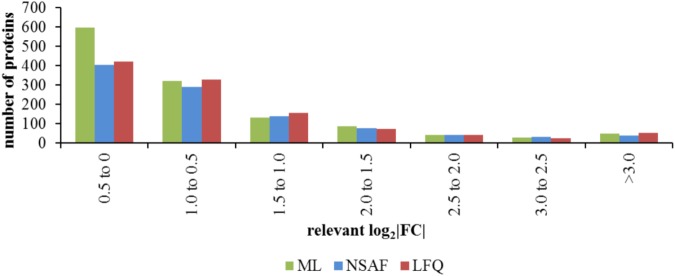
Distribution of protein to different ranges of relevant absolute log_2_FC. The absolute log_2_FC of the three quantitative approaches for the 539 filtered proteins which showed at least one significant change in protein abundance in comparison of the different media, the different growth phases or in that case that the protein abundance was significantly changed by the influence of the growth phase and the media, is shown as sum of the different comparisons.

The calculated log_2_FC obtained by the three quantitative approaches for these 539 proteins were used for analysis of the sensitivity of relative quantification approaches. For this analysis, only the proteins with significantly changed abundance in comparison of the different media or the different growth phases or the influence of the growth phase and medium were selected out of the 539 proteins quantified in all three approaches. A histogram of the absolute log_2_FC obtained for significantly changed proteins by the different approaches revealed a considerably larger group size for the small log_2_FC between -0.5 and 0.5 for the ML approach (**Figure 6**). The distribution of quantified proteins with log_2_FC lower -0.5 as well as greater 0.5 was similar for all quantification approaches. The absolute log_2_FC calculated separately for the different media as well as the different growth phases are represented in **Supplementary Figure S4**.

### Physiological Analysis Based on ML

**FIGURE 7:**
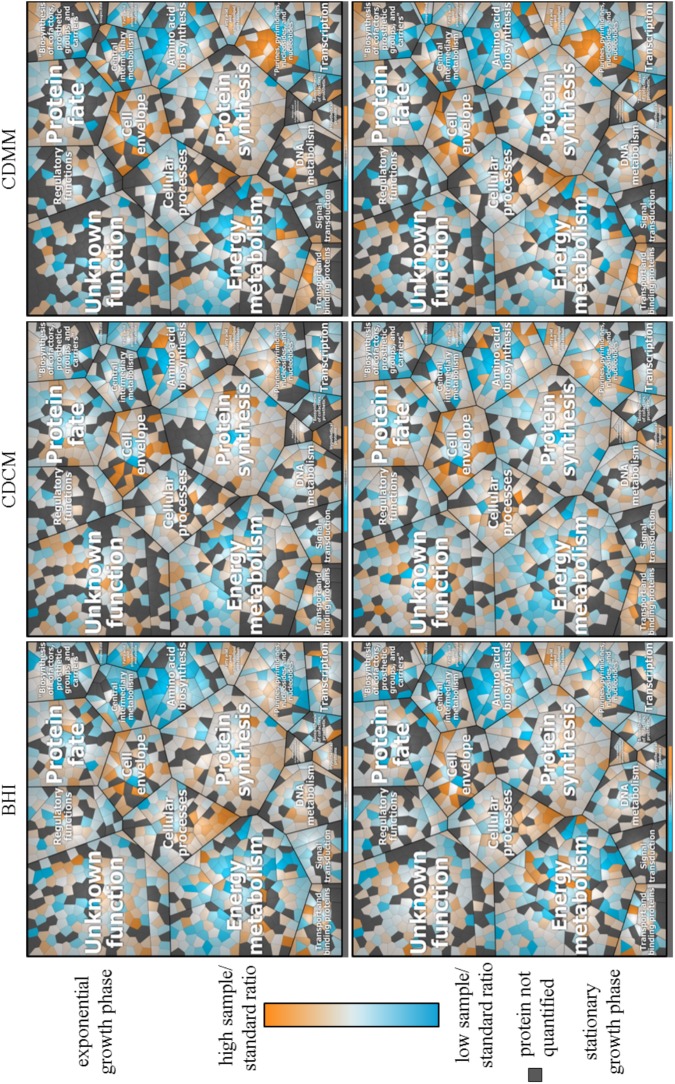
Voronoi treemap of the quantified proteome (1,110 proteins of the 3,781 theoretical proteins) of *C. difficile* 630Δ*erm* assigned to TIGR main roles. Relative sample/standard ratios (high ratio in orange and low ratio in blue) of cytosolic proteins during growth in BHI, CDCM, and CDMM in late exponential growth phase and early stationary growth phase. Proteins were quantified based on ML approach. Proteins not quantified are marked in gray.

The ML approach yielded 1,110 quantified proteins (**Supplementary Table S7**). For the particular media 864, 790, and 728 proteins were quantified in the late exponential growth phase in BHI, CDCM, and CDMM, respectively. In the early stationary growth phase, 844, 894, and 828 proteins were quantified in BHI, CDCM, and CDMM, respectively. Some of these proteins were quantified exclusively in a single growth medium (BHI: 75, CDCM: 77, and CDMM: 38) or exclusively in a single growth phase (late exponential: 77 and early stationary: 114). In the Voronoi treemaps (**Figure 7**) relative protein abundances of the quantified proteins in different media and different growth phase are visualized. For a better visualization a legend of the Voronoi treemaps is shown in **Supplementary Figure S5**. In CDMM for example, proteins of the purine ribonucleotide biosynthesis (PurA, PurB, PurC1, PurC2, PurD, PurE, PurF, PurG, PurH, and PurL) showed a high sample/standard ratio compared to CDCM or BHI media whereas proteins of the V-type ATP synthase (AtpA1, AtpB1, AtpE1, AtpK1) showed a low sample/standard ratio in BHI and CDMM compared to CDCM.

Furthermore, the log_2_FC for CDCM and CDMM were calculated referred to BHI in the current growth phase (**Supplementary Table S7**). The log_2_FC of quantified proteins showed on average 63 proteins with at least 1.5-fold log_2_FC and on average 30 proteins with at least -1.5-fold log_2_FC. Proteins of the aromatic amino acid family (AroF2, CDIF630erm_02622, CDIF630erm_02750), for instance, showed 1.5-fold increase in late exponential and early stationary phase in CDCM and CDMM compared to the sample/standard ratio in BHI cultured cells. EtfA1, EtfA3, EtfB1, and EtfB3 are proteins of the electron transport. The dehydrogenases EtfA1 and EtfB1 showed comparable log_2_FC in CDCM whereas the log_2_FC EtfA3 and EtfB3 were increased in late exponential growth phase and decreased in stationary phase.

## Discussion

In the recent years, a number of quantitative proteome analyses were published for *C. difficile* based on LFQ (Chilton et al., 2014; Otto et al., 2016; Dresler et al., 2017) or chemical labeling (Jain et al., 2011; Chen et al., 2013; Chong et al., 2014; Pettit et al., 2014; Charlton et al., 2015). However, the introduction of a stable isotope into proteins by application of ML enables for accurate quantification of minor changes even after complex and error-prone sample preparation workflows, e.g., by subcellular fractionation (Bantscheff et al., 2007). This would allow studying membrane and surface proteins in a more comprehensive manner facilitating a better understanding of the pathogen thereby offering the chance to identify targets for drug development and therapeutic strategies.

*Clostridioides difficile* is able to grow with amino acids as sole carbon or energy source based on Stickland reaction (Bouillaut et al., 2013). Here, pairs of amino acids are used as electron donor and/or electron acceptor (Bouillaut et al., 2013) and different fermentation products are excreted into the medium during this fermentation reactions (Neumann-Schaal et al., 2015). However, the utilization of amino acids for other purposes than protein biosynthesis hampers the incorporation of heavy nitrogen in the proteins of this pathogen which is a serious challenge for a ML approach in *C. difficile*. This is illustrated by insufficient incorporation rates after 6–8 generations even in media which provides ^15^N-labeled amino acids and ^15^N in all nitrogen sources.

Cultivation of *C. difficile* in CDMM supplemented with casamino acids, cysteine and tryptophan has already been established (Neumann-Schaal et al., 2015; Otto et al., 2016; Riedel et al., 2017). Labeled casamino acids, cysteine, and tryptophan are either very expensive or not even commercially available. Hence, the usage of Celtone as labeled amino acid mixture instead of casamino acids, cysteine, and tryptophan would be an affordable and realizable option for ML in *C. difficile*. Unfortunately, *C. difficile* showed reduced ability to grow in medium with Celtone most probably caused by limitation of nutrients or accumulation of toxic (by-) products. Intensive (and often also expensive) studies on the metabolome and the chemical compositions of the medium during different phases of the labeling process or the development of an alternative culturing procedure could help to increase the incorporation rate. In this study, an inexpensive and functional method for *C. difficile* with stable isotope labeled proteins was established resulting in an incorporation rate of 97.6% on average.

Recent, systematic comparisons of label free and label based protein analysis approaches showed the highest number of protein identifications after applying the label free approach (Patel et al., 2009; Collier et al., 2011; Li et al., 2012; Megger et al., 2014). The authors explained the different number of identified proteins with an increased complexity associated with the reduced total amount of the analyzed material per experimental condition for the labeling approaches (Collier et al., 2011). In this study, the ML and LFQ approaches showed similar numbers of identified protein (2,020 and 2,019) whereas the number of protein identifications for the NSAF approach was slightly reduced (1,788), which demonstrates a lossless identification of proteins during the ML approach. The different numbers within the LFQ approaches in this study can be explained by differences in the algorithms used for database search (SEQUEST versus Andromeda) and different filter criteria (XCorr filtering versus false discovery rate). A previous study with *C. difficile* based on label free 1D gel based LC-MS/MS approach identified 13% less proteins compared to this study, but in that study only cells growing in late exponential growth phase in CDMM and BHI medium were analyzed (Otto et al., 2016).

As described by Bantscheff et al. (2007), the quantification efficiency of a LFQ approach is higher compared to a label based quantification approach as already shown in a comparative study with H9 human embryonic stem cells proteome analyzed with label free (spectral counting) and ML (SILAC) quantification approaches (Collier et al., 2010). These results are in accordance with the number of quantified proteins in the ML approach compared to the LFQ approaches in the presented study. Additionally, the label free approaches resulted in higher proteome coverages compared to a previously study (Otto et al., 2016).

Even though the CVs of the biological replicates showed a distribution over a wider range for the ML approach, the median of 6.78% was lower compared to NSAF and LFQ approaches (**Figure 3**). In previous studies, a CV between biological and technical replicates below 35% were indicated as cutoff for reliable quantification (Maass et al., 2011; Muntel et al., 2014). Moreover, the HCL analysis of biological replicates showed a clear clustering with small tree heights for the ML approach which indicates a low total variation within the data. This is in contrast to the less definite resolution of the clustering for label free approaches with higher tree height (**Figure 4**). The LFQ approach with MaxQuant software indicated a better reproducibility compared to the NSAF approach. This might be caused by the ‘delayed normalization’ in the NSAF approach compared to the LFQ approach with MaxQuant software (Cox et al., 2014). But the reproducibility of the label based quantification approach is even better compared to the LFQ approaches even with small changes in protein abundance between different conditions.

The acquired *R*^2^ values of the correlation analysis were equivalent or even better compared to previous studies comparing label free, iTRAQ-, or SILAC-label based approaches (Patel et al., 2009; Collier et al., 2011; Trinh et al., 2013). Equally, the smaller bootstrap error in the study described here indicates a higher comparability between the label free approaches and showed a smaller deviation between the log_2_FC of the two label free approaches.

Although, less proteins were quantified by the ML approach the number of significantly changed proteins was comparable to this of the NSAF approach. The number of significantly changed proteins by the ML approach was approximately half the number of significantly changed proteins obtained by the LFQ approach (**Table 1**). Furthermore, the number of protein with significant changes of log_2_FC between -0.5 and 0.5 was even higher for the ML approach (**Figure 6**). This indicates an improved sensitivity of the ML approach compared to the LFQ approaches. A comparison of spectral counting versus ML also described an increased number of proteins within the twofold detection range for the ML approach which indicates the overall sensitivity for stable isotope labeling approach (Hendrickson et al., 2009). This results confirms the detection of small changes in protein abundance with an increased accuracy with the ML approach, as previously described (Bantscheff et al., 2007).

The comparison of log_2_FC of the ML approach and a previously described label free protein quantification approach (Otto et al., 2016), resulted in 650 quantified proteins in both approaches for cells grown in CDMM in late exponential growth phase referred to BHI grown cells. Moreover, 156 of these proteins showed an absolute log_2_FC greater than 0.8 in at least the ML approach and furthermore 68% of these proteins were regulated in same direction. Explanations for the 32% differently regulated proteins could be the slightly different harvesting time points (1.0 vs. 0.8). Moreover, it cannot be excluded that, even after many generations in the main culture, proteomic differences are caused by the varying spore preparation protocols. The equally regulated proteins scattered with an *R*^2^ value of 0.64 which indicates a good reproducibility. In detail, proteins of the purine (PurA, PurB, PurC1, PurC2, PurD, PurE, PurF, PurG, PurH, and PurL) and pyrimidine (CarB1, CarB2, PyrB, PyrD, and PyrF) ribonucleotide biosynthesis pathway as well as proteins of the pyridine nucleotides biosynthesis pathway (NadA, NadC, and ThiE1) showed high ^14^N/^15^N ratios in the ML approach. Proteins involved in chemotaxis and motility (FilN2, FliM, and CDIF630erm_01544) showed low sample/standard ratio in the current study. These results were comparable to the analyzed abundance in a previously label free protein quantification approach (Otto et al., 2016). Additionally, relative sample/standard ratios were similar regulated for proteins which fulfill functions, for instance, in amino acid biosynthesis, cellular processes, energy metabolism, protein fate as well as synthesis, or transport and binding proteins according to TIGR main roles. In this study, 5 proteins belonging to pathogenesis TIGR subrole (including ToxA and ToxB) and 15 proteins belonging to toxin production and resistance TIGR subrole were quantified with the ML approach in many of the analyzed cultivation conditions. A closer look on the predicted localization of *C. difficile* proteins showed that in comparison to the previous study from Otto et al. (2016) more proteins from each individual subcellular fraction were identified in our study. With the ML approach, only 91 proteins with predicted cytoplasmic membrane localization could be quantified. It is expected, that an enrichment of this cellular subfraction would lead to a much higher number of membrane bound proteins for which ML provides a suitable quantification strategy.

The pathogenic organism *C. difficile* is the main cause for healthcare-associated diarrhea, which focuses the research more and more onto this pathogen. In previous studies, the analysis of the adaptation processes of this pathogen on the proteome level was carried out based on chemical labeling approaches as well as label free strategies. An adapted cultivation procedure in ^15^N-labeled CDCM enables a successful ML for this pathogen. It is shown that protein quantification based on ML results in a lower number of quantified proteins compared to the LFQ approach; but the quantification is accurate, reproducible and, over all, more sensitive compared to the LFQ strategies. Particularly outstanding is the finding that more proteins with small changes compared to the reference are altered statistically significant. For further applications, such as the analysis of the surface proteome or membrane proteome, the ML can be the better choice due to the error-prone elaborated preparation workflows before MS-analysis which will be reduced due to the internal standard.

## Data Availability

The datasets for this study can be found in the ProteomXchange Consortium via the PRIDE partner repository with the dataset identifier PXD010279.

## Author Contributions

AT-S, AO, and DB developed and discussed the experimental design. AT-S did the laboratory work and produced the proteome data. AT-S, SM, KP, and AO analyzed the proteome data and all authors discussed the data. AT-S, SM, and DB conceived and wrote the manuscript with support from AO and KP.

## Conflict of Interest Statement

The authors declare that the research was conducted in the absence of any commercial or financial relationships that could be construed as a potential conflict of interest.

## Footnotes

1 https://proteome.gs.washington.edu/software/IDCalc/

## Abbreviations

BHI: brain heart infusion
CDCM: *C. difficile* Celtone medium
CDI: *C. difficile* infection
CDMM: *C. difficile* minimal medium
CV: coefficient of variation
HCL: hierarchical clustering
iTRAQ: isobaric Tags for Relative and Absolute Quantification
LFQ: label free quantification
log_2_FC: logarithmic fold change to base 2
ML: metabolic labeling
MS: mass spectrometry
NSAF: normalized spectral abundance factor
OD: optical density
*R*^2^: coefficient of determination
SD: standard deviation
SILAC: stable isotope labeling by amino acids in cell culture
TMT: Tandem Mass Tag
