# Supplementary material for: A Metabolic Labeling Strategy for Relative Protein Quantification in Clostridioides difficile
Source: Front Microbiol. 2018 Oct 16;9:2371. doi: 10.3389/fmicb.2018.02371 (PMC6198727; doi:10.3389/fmicb.2018.02371)
Supplement: Supplementary file 1 [file Table_1.docx]

**Table S1**: *C. difficile* minimal medium (CDMM) and *C. difficile* Celtone medium (CDCM). A 1-liter volume of CDMM was made by filling 200 ml amino acids (5x), 100 ml salts (10x), 10 ml trace salts (100x), 10 ml glucose (100x), 5 ml vitamins (200x) and 2 ml iron (500x) up to 1 liter with sterile *aq. bidest*. For CDCM the amino acids mixture of casamino acids (Carl Roth, Germany), L-tryptophan and L-cysteine were replaced by 50 ml Celtone. Each stock solution was sterilize-filtered (0.22 µm pore size). *For ^15^N-CDCM this ingredient were used as ^15^N labelled (98%) ingredient.

| **Stock solution name** | **Stock solution component** | **Concentration in stock solution** | **Final concentration in CDMM** | **Final concentration in CDCM** |
| --- | --- | --- | --- | --- |
| Amino acids (5x) | Casamino acids  L-tryptophan  L-cysteine | 50 g/l  0.5 g/l  2.5 g/l | 10 g/l  0.1 g/l  0.5 g/l | -  -  - |
| Celtone (20x) | Celtone* | 50 g/l | - | 2.5 g/l |
| Salts (10x) | Na_2_HPO_4_  NaH_2_PO_4_ x H_2_O  KH_2_PO_4_  NaCl | 50 g/l  20 g/l  9 g/l  9 g/l | 5 g/l  2 g/l  0.9 g/l  0.9 g/l | 5 g/l  2 g/l  0.9 g/l  0.9 g/l |
| Sugar (100x) | Glucose | 200 g/l | 2 g/l | 2 g/l |
| Trace salts (100x) | (NH_4_)_2_SO_4_*  CaCl_2_ x 2 H_2_O  MgCl_2_ x 6 H_2_O  MnCl_2_ x 4 H_2_O  CoCl_2_ x 6 H_2_O  NaHSeO_3_ | 4 g/l  2.6 g/l  2 g/l  1 g/l  0.1 g/l  15 mg/l | 40 mg/l  26 mg/l  20 mg/l  10 mg/l  1 mg/l  0.015 mg/l | 40 mg/l  26 mg/l  20 mg/l  10 mg/l  1 mg/l  0.015 mg/l |
| Iron (500x) | FeSO_4_ x 7 H_2_O | 2 g/l | 4 mg/l | 4 mg/l |
| Vitamins (200x) | D-biotin  Ca-D-pantothenate  Pyridoxine | 60 mg/l  0.2 g/l  0.2 g/l | 0.3 mg/l  1 mg/l  1 mg/l | 0.3 mg/l  1 mg/l  1 mg/l |
