# Supplementary figures and images for: A Metabolic Labeling Strategy for Relative Protein Quantification in Clostridioides difficile
Source: Front Microbiol. 2018 Oct 16;9:2371. doi: 10.3389/fmicb.2018.02371 (PMC6198727; doi:10.3389/fmicb.2018.02371)

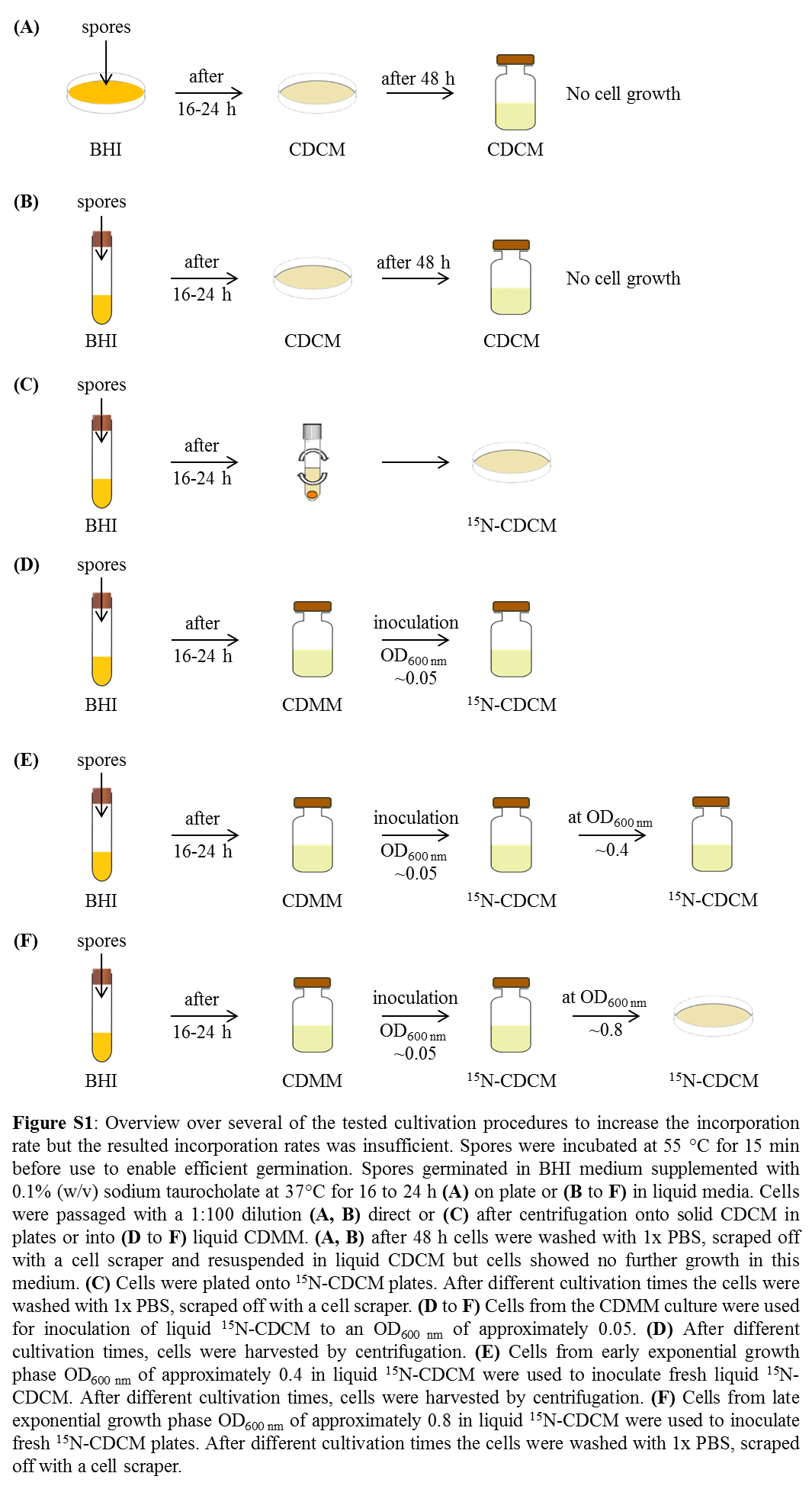

Supplement: Supplementary file 8 [file Image_1.TIF]

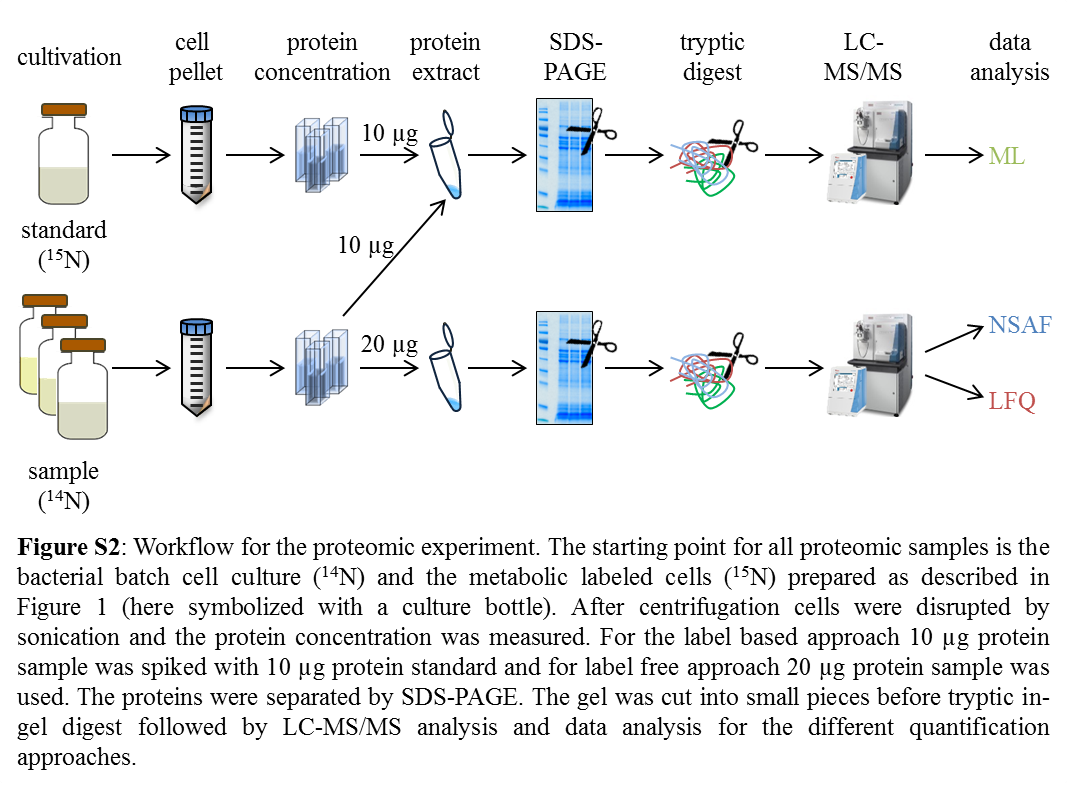

Supplement: Supplementary file 9 [file Image_2.TIF]

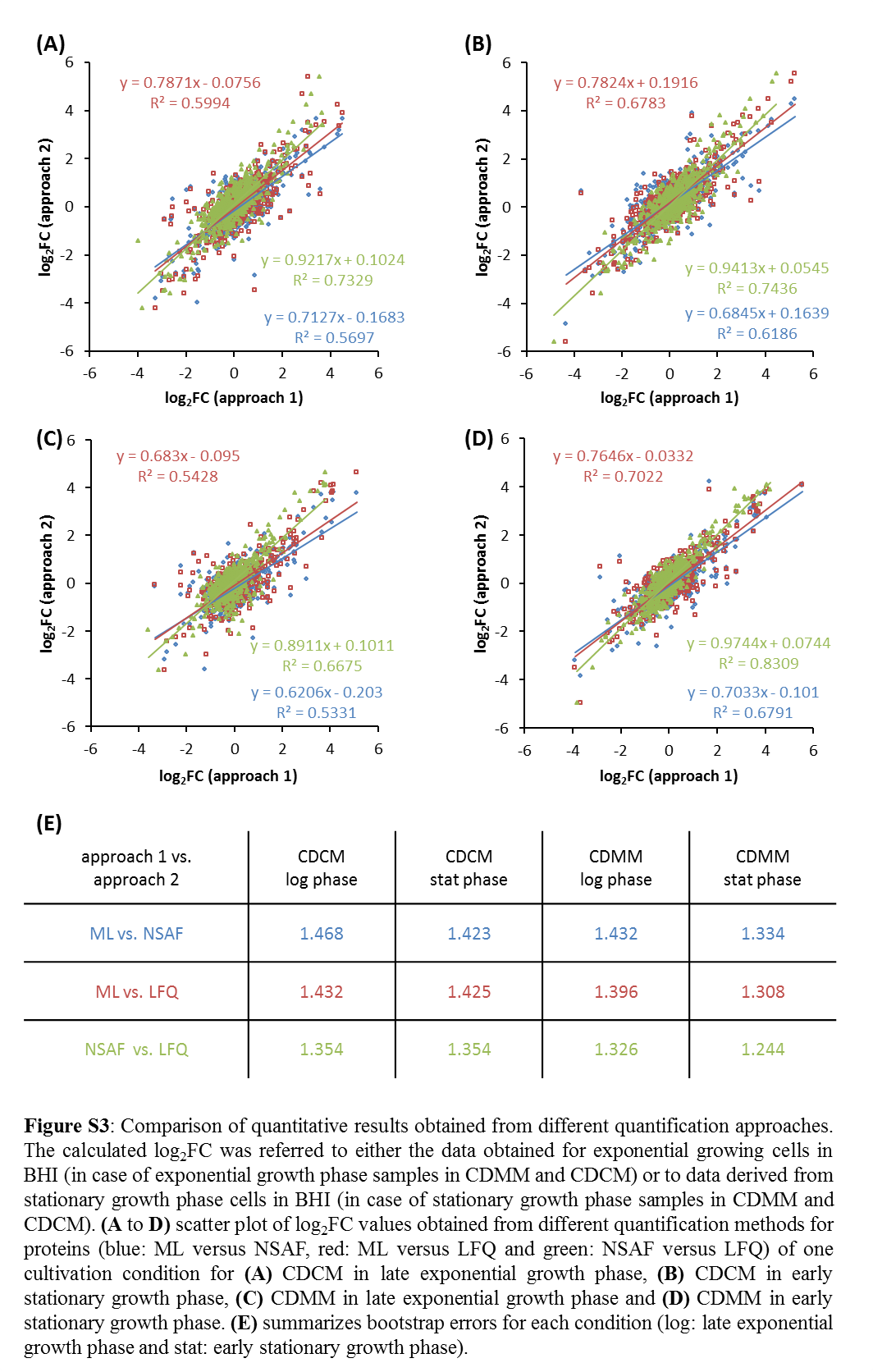

Supplement: Supplementary file 10 [file Image_3.TIF]

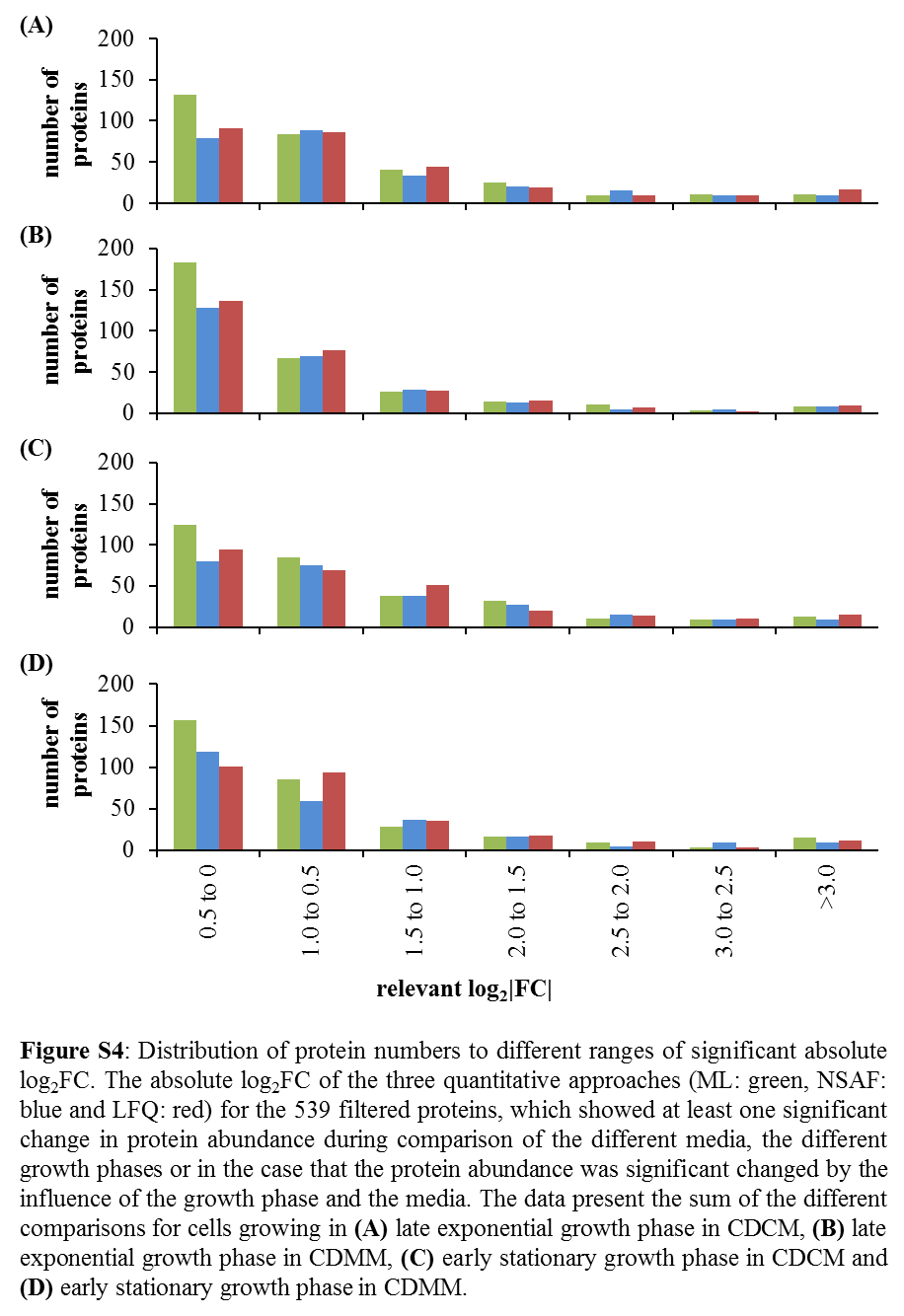

Supplement: Supplementary file 11 [file Image_4.TIF]

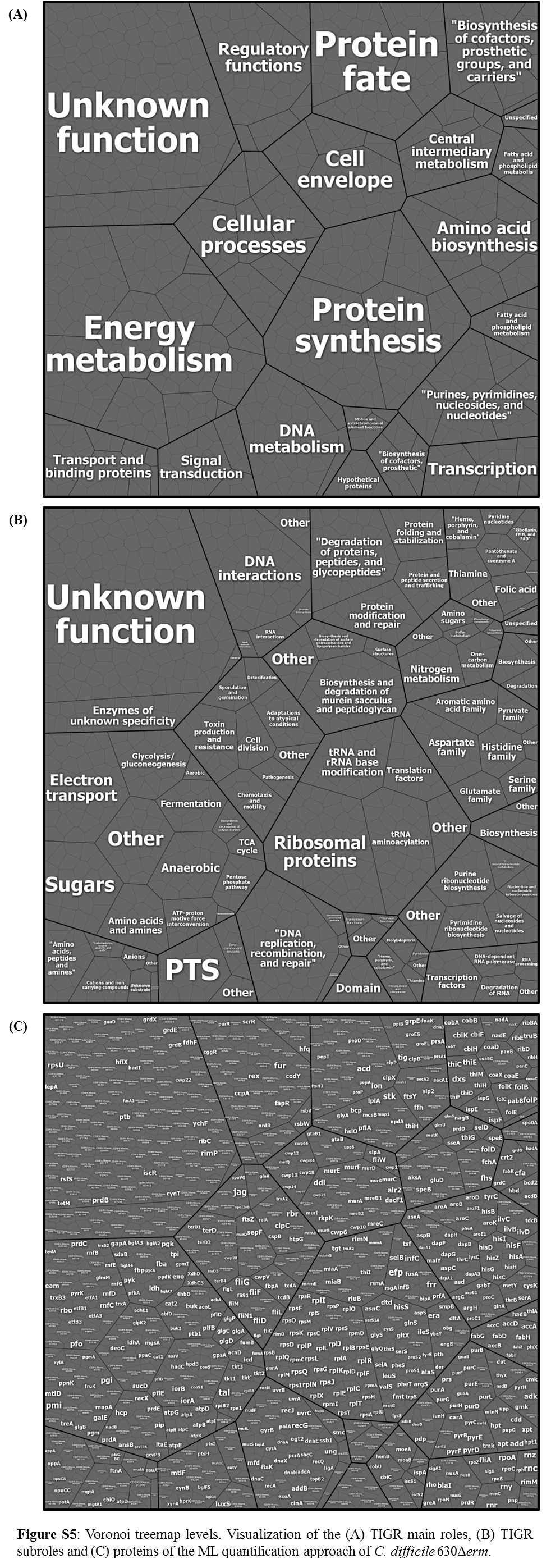

Supplement: Supplementary file 12 [file Image_5.TIF]
